# Hyperglycemia during the immediate period following liver transplantation

**DOI:** 10.4155/fsoa-2015-0010

**Published:** 2016-01-27

**Authors:** K Tuesday Werner, Patricia A Mackey, Janna C Castro, Elizabeth J Carey, Harini A Chakkera, Curtiss B Cook

**Affiliations:** 1 Division of Gastroenterology & Hepatology, Mayo Clinic Hospital, Phoenix, AZ 85054, USA; 2 Division of Endocrinology, Mayo Clinic, 13400 East Shea Boulevard, Scottsdale, AZ 85259, USA; 3 Information Technology, Mayo Clinic Hospital, Phoenix, AZ 85054, USA; 4 Division of Nephrology, Mayo Clinic Hospital, Phoenix, AZ 85054, USA; 5 Division of Preventive, Occupational & Aerospace Medicine, Mayo Clinic, Scottsdale, AZ 85259, USA

**Keywords:** diabetes mellitus, hospital, hyperglycemia, inpatient, liver transplant

## Abstract

**Aim:**

High blood glucose levels in the hospital are common among transplant recipients.

**Methods:**

Retrospective analysis, stratified by diagnosis of pretransplant diabetes mellitus (DM).

**Results:**

Of 346 patients, 96 had pretransplant DM (insulin, n = 60; no insulin, n = 36) and 250 did not. Patients with pretransplant DM had higher inpatient mean glucose levels and more hyperglycemia and hypoglycemia (all p < 0.01). For patients without pretransplant DM, the need for insulin at discharge increased 23% for every 5-year age increase (odds ratio: 1.23; 95% CI: 1.06–1.44; p = 0.007) and 51% for every five units of glucose measurements >180 mg/dl (OR: 1.51; 95% CI: 1.23–1.95; p < 0.01).

**Conclusion:**

Inpatient hyperglycemia was common in liver transplant recipients. Hospital practitioners must anticipate the need to teach self-management skills to liver transplant recipients.

## Abstract (lay abstract)

**Lay abstract:** High blood glucose levels (also known as hyperglycemia) in the hospital are common among patients who have received a transplant. The authors conducted a study to determine how often high blood glucose values occurred in patients who received a liver transplant and found that values were highest in people who had diabetes before the transplant. However, even patients who did not have a history of diabetes had hyperglycemia and needed insulin treatment. Providers caring for these patients in the hospital must be prepared to provide education in diabetes self-management skills to virtually all patients undergoing a liver transplant.

**Figure 1.**
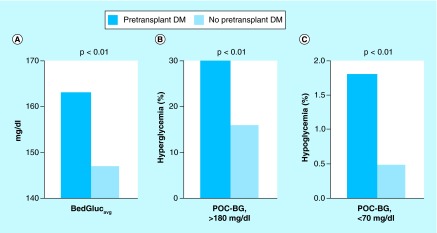
**Comparison of glucose levels according to pretransplant diabetes mellitus status.** **(A)** Patient-stay mean glucose (BedGluc_avg_). **(B)** Frequency of hyperglycemia. **(C)** Frequency of hypoglycemia between persons with and without pretransplant DM. DM: Diabetes mellitus; POC-BG: Point-of-care blood glucose.

**Figure 2.**
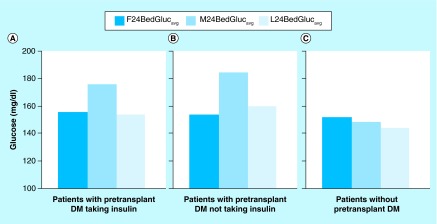
**Mean point-of-care glucose values for the first 24 h (F24BedGluc_avg_), the midpoint (M24BedGluc_avg_) and the last 24 h (L24BedGluc_avg_) of the hospital stay following liver transplant, according to pretransplant status of diabetes mellitus.** See text for values and statistical comparisons. DM: Diabetes mellitus.

**Figure 3.**
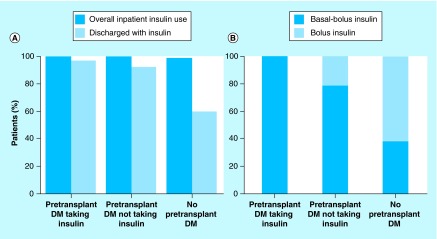
**Use of inpatient insulin in liver transplant patients, according to pretransplant diabetes mellitus status.** **(A)** Percentage of patients receiving insulin during the hospital stay and the proportion discharged on insulin therapy. **(B)** Type of insulin regimen used during the hospital stay. DM: Diabetes mellitus.

Hyperglycemia is a well-described complication following receipt of a solid organ transplant [1–4]. Solid organ transplant recipients with diabetes mellitus (DM) who are seen in the outpatient setting after hospital discharge typically either had DM pretransplant or DM following transplant. The latter situation is often referred to as new-onset diabetes after transplant (NODAT). NODAT is a strong predictor of graft failure and cardiovascular death in the solid organ transplant population [1–4]. Potential risk factors associated with NODAT include the conventional risk factors for Type 2 DM, such as race/ethnicity, obesity, family history of DM, increasing age and impaired glucose tolerance pretransplant, but also include use of immunosuppressive medications [1–5]. Among liver transplant recipients, other implicated risk factors include hepatitis C virus infection and receipt of organs from deceased donors [4,6].

Regarding glucose metabolism in solid organ transplant recipients, most studies have focused on NODAT. However, hyperglycemia occurring during the immediate post-transplant period – while the patient is still hospitalized – also has implications for outcome. For instance, in a prior analysis of patients after renal transplant, the prevalence of inpatient hyperglycemia was high even among those without known DM, and inpatient hyperglycemia was associated with NODAT development [7,8]. Among liver transplant recipients, intraoperative and postoperative hyperglycemic events have been associated with death and allograft rejection and effective hyperglycemia management can reduce rates of postoperative infections [9–11]. Therefore, earlier identification of hyperglycemia in the inpatient setting, and optimization of inpatient glycemic control may have clinical implications for long-term patient and graft survival among patients undergoing liver transplant.

Additionally, for the liver transplant population, having more data on the burden of inpatient hyperglycemia is important because considerable staff resources may be required to provide education to patients about blood glucose self-monitoring, insulin self-administration and dietary management before discharge. Allowing sufficient time to provide such education is key to ensuring a timely discharge. In addition, standards of care relating to management of inpatient hyperglycemia and DM are available and apply equally to patients receiving any solid organ transplant [12–15]. No published data are available on the prevalence or patterns of inpatient hyperglycemia or how hyperglycemia is managed during inpatient care after liver transplant. We conducted a retrospective analysis to better characterize inpatient hyperglycemia and assess the pharmacologic treatment of patients undergoing a first-time liver transplant.

## Methods

### Case selection

For the present study, the immediate post-transplant period was defined as beginning the day after completion of the transplant procedure to time of discharge. The analysis included all adult patients undergoing a first-time solitary liver transplant between 1 January 2007 and 31 December 2012. Patient data were obtained through a combination of chart review and data retrieval from laboratory and pharmacy information systems. Available data included demographic characteristics (e.g., age, sex, race/ethnicity), whether a DM diagnosis existed before transplant, cause of end-stage liver disease (ESLD) and type of outpatient DM therapy before receipt of transplant, if applicable. Patients with pretransplant DM were subcategorized by insulin treatment and no insulin treatment (i.e., DM patients treated with oral agents or diet), as previously described for the renal transplant population [7].

### Postoperative management after liver transplant

A standardized immunosuppression protocol of corticosteroids, tacrolimus and mycophenolate mofetil is used postoperatively. Tacrolimus dose is adjusted to achieve a trough level of 8–10 ng/ml for the first 21 days after transplant. Mycophenolate mofetil therapy (1000 mg twice daily) is started at the time when oral food intake resumes. Corticosteroid administration follows the regimen of 500 mg intravenous methylprednisolone acetate preoperatively, 50 mg twice daily on postoperative day 0 and 25 mg twice daily on postoperative day 1. Next, 20 mg oral prednisone twice daily is administered on postoperative days 2 and 3, 15 mg on postoperative days 4–6 and 10 mg twice daily on postoperative days 7–10, followed by progressive tapering to a treatment stop at 4 months postoperatively.

### Assessment of inpatient glycemic control

Point-of-care blood glucose (POC-BG) measurements were used to assess glycemic control. They were obtained using standardized instrumentation (Accu-Chek Inform; Roche Diagnostics). POC-BG data were used to calculate the patient-stay mean glucose level (BedGluc_avg_), and also the mean glucose during the first 24 h following transplant (F24BedGluc_avg_), during the last 24 h before discharge (L24BedGluc_avg_) and at the midpoint between the 2 (M24BedGluc_avg_), as has been done previously [7,16]. The per-patient frequency of hyperglycemic and hypoglycemic events was calculated by dividing the number of their glucose measurements >180 mg/dl and <70 mg/dl by their number of total POC-BG measurements performed between the day of transplant and the day of discharge. Where available, hemoglobin A_1c_ was recorded.

### Overview of hyperglycemia treatment

The inpatient endocrinology service was consulted routinely to assist with hyperglycemia management and provision of education in both self-monitoring of blood glucose and insulin administration. The goal was to keep glucose values between 140 and 180 mg/dl, the currently recommended target range of inpatient glycemic control [12–15]. Insulin infusions were ordered during the first 24 h following surgery and used at variable rates when needed, followed by conversion to a subcutaneous insulin regimen. A previously published algorithm for managing hyperglycemia in postoperative inpatients with and without pre-existing DM was used [17]. Briefly, basal-bolus insulin therapy was recommended for patients already taking insulin as outpatients or with two glucose values >180 mg/dl within a 24-h period. An initial dose of 0.5 units/kg body weight was initiated and divided equally among basal and prandial insulin if the patient was eating. Insulin doses were adjusted to keep glucose levels in the target range above and to avoid hypoglycemia.

### Definitions of inpatient insulin regimen

In general, a basal-bolus regimen (e.g., long-acting insulin at bedtime plus rapid-acting insulin with meals and correction doses as needed for hyperglycemia) is the most effective insulin management for hospitalized patients with hyperglycemia, including in postoperative patients [12–15,18]. Insulin was the sole therapy utilized in these patients when pharmacological therapy was needed to treat hyperglycemia. Assistance with insulin therapy was provided by the inpatient endocrinology consult team. In the present study, the type of insulin therapy administered was determined by linking clinical data with the pharmacy information system. Long-acting insulin therapy (glargine or NPH in our hospital) was termed basal and rapid- or short-acting insulin was classified as bolus if provided as a prandial dose or a correction dose or both. As previously described for the postoperative patient, patterns of insulin administration were designated as none, bolus only or basal-bolus; any use of premixed insulin was grouped under basal-bolus [16,19,20].

### Data analysis

Analyses were conducted similar to what was described for the inpatient assessment of postrenal transplant patients [7,8]. Comparisons were made according to the history of a pretransplant DM diagnosis. In addition, the subset of patients with pretransplant DM was compared according to whether they received outpatient treatment with insulin. Mean POC-BG levels, frequency of hyper- and hypo-glycemia and insulin use were determined and compared. For patients without a history of pretransplant DM, potential factors associated with being discharged on insulin therapy were evaluated through a logistic regression analysis incorporating age, sex, race/ethnicity, BMI, cause of ESLD (viral vs nonviral) and the number of POC-BG measurements >180 mg/dl. Differences in categorical variables were evaluated with χ^2^ test and continuous variables with t test. Data are either percent for categorical variables and mean (SD) for continuous variables.

## Results

### Patient characteristics

Between January 2007 and December 2012, 346 first-time liver transplants were performed (Table 1). Of these transplants, 96 patients (28%) had a diagnosis of pretransplant DM and 250 (72%) did not. Among the 96 patients with pretransplant DM, 60 (63%) were treated with insulin and 36 (37%) with diet alone or oral agents. The majority of DM patients were men (67%) and of white race/ethnicity (84%). The dominant donor type in patients with and without DM was cadaveric. Compared with all patients with pretransplant DM, patients without pretransplant DM were slightly younger (p = 0.04) and had a higher BMI (p = 0.04). In addition, a greater proportion of patients without pretransplant DM received a liver transplant from a living donor (p < 0.01). The distribution of ESLD cause also differed (p < 0.01) between the patient group with and the group without pretransplant DM; no differences were detected in the other demographic variables (Table 1). A difference was detected in BMI (p = 0.049), but not in age, sex, race/ethnicity, ESLD cause or donor type (cadveric vs living) between the two DM subpopulations on the basis of pretransplant DM therapy. A higher, but not significant (p = 0.05), hemoglobin A_1c_ level was found among patients with pretransplant DM who were taking insulin (Table 1; p-values not shown). No differences in length of stay were seen following transplant.

### Glycemic control

All patients with pretransplant DM and 90% of those without pretransplant DM had hyperglycemia in the immediate post-transplant period. Slightly more glucose measurements were performed per day in patients with pre-existing DM (5 [3]) than in persons without DM (4 [2]) (p < 0.01), but did not differ among patients with pre-existing DM according to outpatient insulin use (p = 0.77). The BedGluc_avg_ was higher in patients with pretransplant DM than in patients without it (163 [21] mg/dl versus 147 [17] mg/dl, p < 0.01) (Figure 1A). In addition, patients with pretransplant DM had a greater proportion of POC-BG measurements >180 mg/dl during their hospital stay (30% [15%] vs 16% [14%], p < 0.01) (Figure 1B). Although hypoglycemia was uncommon in both groups, patients with pretransplant DM had a higher percentage of glucose measurements <70 mg/dl than those who did not (1.8% [3.9%] vs 0.5% [1.5%], p < 0.01) (Figure 1C). No differences were detected in these three glucose parameters between DM patients on the basis of outpatient insulin therapy (all p ≥ 0.23, data not shown).

Changes in glycemic control across the post-transplant inpatient period were next examined, as assessed by calculating and comparing values of F24BedGluc_avg_, M24BedGluc_avg_ and L24BedGluc_avg_. For patients with pretransplant DM who were taking insulin, glycemic control deteriorated between the initial 24 h and the middle 24 h of hospital stay, increasing from a mean (SD) glucose level of 156 (30) mg/dl to 176 (56) mg/dl (p < 0.01) (Figure 2A). Glycemic control then improved, with mean glucose decreasing from the midpoint to 154 (38) mg/dl by the last 24 h of hospital stay (p < 0.01), such that there was no difference between F24BedGluc_avg_ and L24BedGluc_avg_ values (p = 0.88). A similar pattern was seen for patients with pretransplant DM who were not treated with insulin as outpatients (Figure 2B). For these patients with pretransplant DM not taking insulin, the mean (SD) F24BedGluc_avg_ was 154 (28) mg/dl versus 185 (52) mg/dl for M24BedGluc_avg_ (p < 0.01), and the mean (SD) L24BedGluc_avg_ was 160 (40) mg/dl (p = 0.04 compared with M24BedGluc_avg_). Comparison of mean glucose levels between corresponding time points from the two subsets of DM patients showed no differences (all p > 0.45).

For cases without a history of pretransplant DM, a different hyperglycemia pattern was detected during the post-transplant inpatient period (Figure 2C): Deteriorating glycemic control was not seen during the midpoint of the hospital stay. Mean (SD) F24BedGluc_avg_, M24BedGluc_avg_ and L24BedGluc_avg_ values were 152 (29) mg/dl, 148 (31) mg/dl and 144 (28) mg/dl, respectively. F24BedGluc_avg_ and M24BedGluc_avg_ values were comparable (p = 0.20). The mean L24BedGluc_avg_ was not significantly lower than the M24BedGluc_avg_ (p = 0.05), but the L24BedGluc_avg_ was significantly less than the F24BedGluc_avg_ (p < 0.01).

### Inpatient insulin regimen

Insulin was administered to all patients with pretransplant DM during their total hospital stay, regardless of prior history of insulin therapy. With the exception of one patient, insulin was also administered to all patients who did not have a history of pretransplant DM (Figure 3A). At the time of discharge, insulin therapy was provided to 97% of pretransplant DM patients on outpatient insulin therapy, 92% of pretransplant DM cases who were not taking insulin and 59% of patients who did not have pretransplant DM. Insulin regimens at discharge differed according to pretransplant DM history (Figure 3B). Of patients with pretransplant DM who had insulin therapy before operation, 100% required basal-bolus insulin to control hyperglycemia. For patients with pretransplant DM who were not treated with insulin as outpatients, 78% needed the basal-bolus regimen and 22% received bolus alone. Finally, for patients without a pretransplant DM history, only 38% were treated with basal-bolus insulin therapy and 61% received bolus insulin only.

Total daily units of insulin delivered were calculated for the first, middle and last 24-h periods corresponding to the glucose levels. Changes in total daily amount of insulin were consistent with the changes in mean glucose levels at these time points, with more insulin administered during the periods of highest mean glucose levels for patients with pretransplant DM. For patients with pretransplant DM who were taking insulin before surgery, mean (SD) units of insulin administered were 57 (47), 86 (66) and 59 (45) during the first, middle and last 24 h of hospital stay. For patients with DM who were not receiving insulin as outpatients, the corresponding mean (SD) insulin amounts were 35 (31), 58 (57) and 43 (39) units. Lastly, patients without pretransplant DM were given an average 15 (17), 23 (30) and 17 (21) units of insulin during the first, middle and last 24-h periods of hospital stay.

### Variables associated with needing insulin at discharge among non-DM patients

A patient without a history of pretransplant DM may be overwhelmed about the possibility of needing insulin therapy after hospital discharge, and the associated need to perform self-monitoring of blood glucose adds to an already complicated post-transplant medication regimen and recovery period. To better inform the patient and the inpatient care team, we conducted a logistic regression for evaluation of variables associated with the probability of hospital discharge with insulin in this patient group (Table 2). The need for insulin at discharge increased 23% for every 5-year increase in age (odds ratio [OR]: 1.23; 95%CI: 1.06–1.44; p = 0.007) and increased 51% for every five POC-BG units >180 mg/dl (OR: 1.51; 95%CI: 1.23–1.95; p < 0.01). BMI, race/ethnicity, sex and cause of ESLD were not associated.

## Discussion

In terms of the importance of inpatient glucose control, it is well recognized that inpatient hyperglycemia is associated with a number of adverse outcomes postoperatively [20]. Management of inpatient hyperglycemia in the period immediate after liver transplantation should be viewed within the same context of current guidelines regarding the importance of controlling hospital hyperglycemia in any patient postoperatively, yet little data exist about hospital-based management of hyperglycemia in the postoperative liver transplant population. Moreover, the inpatient setting can afford opportunities to diagnose and treat hyperglycemia and to deliver education in self-management skills that the patient needs following the transition to the outpatient setting. Using the prior analysis of the postrenal transplant population [7,8] as a model, we undertook a retrospective analysis of first-time liver transplant recipients, to better define the characteristics and treatment of this population with regard to hyperglycemia in the immediate post-transplant period. To our knowledge, this is the first detailed analysis of inpatient glycemic control and therapy in this population.

The performed analyses indicate that the prevalence of inpatient hyperglycemia was high not only in persons with known pretransplant DM, but also among individuals without pre-existing DM. However, the patients with pretransplant DM had higher BedGluc_avg_ and more frequent hyperglycemia than the patients without pretransplant DM. The degree of hyperglycemia was similar among the DM patients regardless of whether insulin was used pretransplant. In those with pretransplant DM, transient worsening of hyperglycemia occurred by the midpoint of the post-transplant period; this change was not noted in patients without known DM.

Patterns of insulin use in this hospitalized liver transplant population were elucidated. Although persons treated with insulin as outpatients may be expected to need insulin while hospitalized, we found that all patients with pretransplant DM who did not previously receive insulin therapy also required it while hospitalized. In addition, a substantial proportion of patients without known DM received insulin during their hospital stay. Most patients required basal-bolus insulin to control hyperglycemia, although specific regimens differed according to DM history. All patients with pretransplant DM who were previously taking insulin were administered basal-bolus insulin as inpatients. Some patients with known DM but who were not taking insulin as outpatients were able to be treated with bolus insulin alone, although most of these patients also needed basal-bolus therapy. Predominantly, rapid-acting insulin was used to manage hyperglycemia in patients without pretransplant DM. This approach may be appropriate, given their less frequent and less severe hyperglycemia. The amount of insulin administered corresponded to each period examined, with greater doses delivered during the periods of highest mean glucose levels for those with know DM. However, these data should be interpreted with caution because insulin requirements (and subsequent dosing) can change daily and are not driven solely by glucose levels but also by changes in corticosteroid doses, nutritional support and acuity status. Hence, cause and effect cannot be assumed.

The findings described herein have implications regarding the resources needed for inpatient DM education services. All patients with pretransplant DM who did not take insulin therapy previously will most likely need a review of blood glucose self-monitoring and insulin administration before discharge. Knowing this information, perhaps introduction of such education in the pretransplant outpatient setting (rather than introducing it in the acute post-transplant hospital setting) could facilitate inpatient care. Moreover, we found that 59% of the liver transplant recipients without pretransplant DM were discharged with insulin, and they would have required training in a whole new set of skills to monitor and manage their hyperglycemia before their transition to outpatient care.

These observations are similar to what has been noted for renal transplant patients [7]. In this analysis, only two variables – age and hyperglycemia frequency – were associated with the need for insulin at discharge among patients without pretransplant DM. No guidelines or criteria are available for determining whether a patient is to be sent home with insulin after liver transplant. However, practitioners consider many variables and criteria when making the decision to discharge a patient on insulin. These include ongoing use of corticosteroids, insulin requirements in the hospital setting and especially nearer to the time of discharge to maintain glucose levels in the target range, and a history of pre-existing DM and insulin use. Because it cannot be known at hospitalization what proportion of patients without pretransplant DM will resolve their hyperglycemia or will continue to require insulin after discharge, all these patients need ongoing education and support regarding hyperglycemia management.

The present analysis has some limitations. This review of electronic records was retrospective and some variables, such as DM type, were not available. Although some parallels were found to the hospitalized renal transplant population, our results cannot be generalized to patients who receive other types of transplants (e.g., heart transplant, bone marrow transplant) either in this institution or at other hospitals. Finally, provider behavior, such as the reasons for choosing a particular insulin regimen, cannot be determined from the data.

## Conclusion

Despite the limitations, this analysis, together with the prior analysis focusing on the immediate post-transplant period among renal transplant population [7], expands the understanding of inpatient hyperglycemia and its management for patients undergoing solid organ transplant. A majority of liver transplant recipients who had pre-existing DM required insulin as inpatients and at discharge. In addition, a substantial number of persons without pretransplant DM had hyperglycemia and required insulin during the hospital phase of their care and had to be discharged on insulin. Inpatient care teams must be prepared to provide education in DM self-management skills to virtually all patients undergoing a liver transplant. Adoption of appropriate lifestyle modifications and adherence to a healthy meal plan to optimize glycemic control postoperatively will need to be emphasized to these patients.

## Future perspective

Further study is needed to establish which variables predict inpatient hyperglycemia and the need for insulin therapy to better guide practitioners on how to treat these patients. Among patients with DM who had no history of outpatient insulin use and in those who had no history of DM, long-term follow-up is required to determine which patients recover from their hyperglycemic episode and can have insulin therapy withdrawn. More studies in the liver transplant population are needed to determine variables associated with the need for ongoing hyperglycemia care as outpatients so that resources can be managed appropriately. Finally, it would be of interest to determine whether aggressive lifestyle changes in the outpatient setting before transplant could modify the severity of inpatient hyperglycemia.

**Table 1.** **Demographics of patients undergoing liver transplant, according to diabetes mellitus status.**

	**Pretransplant DM**			**No DM pretransplant (n = 250)**	**p-value^†^**
**Characteristic**	**All patients (n = 96)**	**Insulin (n = 60)**	**Diet or oral agents (n = 36)**		
Age, mean (SD), years	56 (9)	56 (10)	56 (8)	54 (9)	0.04
Male sex	64 (67)	42 (65)	25 (69)	168 (67)	0.93
White race	81 (84)	50 (83)	31 (86)	220 (88)	0.38
BMI, mean (SD), kg/m^2^	29.8 (6.6)	28.7 (5.9)	31.6 (7.4)	28.2 (5.6)	0.04
HbA_1c_, mean (SD),%	6.2 (1.2)	6.4 (1.3)^‡^	5.8 (1.0)^§^	NA	
Length of stay following transplant, mean (SD), days	13 (12)	14 (14)	12 (9)	12 (10)	0.50
Cause of ESLD:					<0.01
– Alcohol	20 (21)	10 (17)	10 (28)	38 (15)	
– Autoimmune disease	6 (6)	3 (5)	3 (8)	44 (18)	
– Cryptogenic/other	14 (15)	11 (18)	3 (8)	25 (10)	
– NASH	16 (17)	9 (15)	7 (19)	17 (7)	
– Viral infection	40 (42)	27 (45)	13 (36)	126 (50)	
Cadaveric donor	86 (90)	52 (87)	34 (94)	195 (78)	<0.01

Values are presented as number and percentage of patients unless specified otherwise.

^†^Comparing pretransplant DM versus no pretransplant DM.

^‡^Data available in 43 patients.

^§^Data available in 24 patients.

DM: Diabetes mellitus; ESLD: End-stage liver disease; HbA_1c_: Hemoglobin A_1c_; NA: Not available; NASH: Nonalcoholic steatohepatitis.

**Table 2.** **Variables associated with being discharged on insulin therapy (versus no insulin therapy) in liver transplant patients without a pretransplant history of diabetes mellitus.**

**Characteristic**	**OR (95% CI)**	**p-value**
Age (years)^†^	1.22 (1.04–1.44)	0.01
POC-BG >180 mg/dl^‡^	1.51 (1.22–1.95)	<0.01
BMI (kg/m^2^)	1.02 (0.98–1.08)	0.30
White race (vs nonwhite race)	1.31 (0.55–3.15)	0.54
Male sex (vs female sex)	1.04 (0.57–1.88)	0.89
Cause of ESLD (viral vs nonviral)	1.09 (0.62–1.94)	0.76

^†^Per 5-year increase.

^‡^Per 5-unit increase.

ESLD: End-stage liver disease; OR: Odds ratio; POC-BG: Point-of-care blood glucose.

**Executive summary**

Inpatient hyperglycemia is common among liver transplant recipients, even among persons without preexisting diabetes.
Mean glucose levels and frequency of hyperglycemia were highest in liver transplant recipients who had pre-existing diabetes, and they will have to be treated more aggressively than for patients who did not have diabetes before transplant.
Many patients without pretransplant diabetes had to be discharged with insulin treatment.
Inpatient care teams must be prepared to provide education in glucose monitoring, insulin administration and nutrition therapy to virtually all patients receiving a liver transplant.
